# An Approach to Identifying Spatial Variability in Observed Infectious Disease Spread in a Prospective Time-Space Series with Applications to COVID-19 and Dengue Incidence

**DOI:** 10.21203/rs.3.rs-3859620/v1

**Published:** 2024-01-24

**Authors:** Chih-Chieh Wu, Chien-Hsiun Chen, Shann-Rong Wang, Sanjay Shete

**Affiliations:** 1 Department of Environmental and Occupational Health, College of Medicine, National Cheng Kung University, Tainan, Taiwan; 2 Department of Statistics, College of Management, National Cheng Kung University, Tainan, Taiwan; 3 Institute of Biomedical Sciences, Academia Sinica, Taipei, Taiwan; 4 Department of Biostatistics, The University of Texas MD Anderson Cancer Center, Houston, Texas, USA

## Abstract

Most of the growing prospective analytic methods in space-time disease surveillance and intended functions of disease surveillance systems focus on earlier detection of disease outbreaks, disease clusters, or increased incidence. The spread of the virus such as SARS-CoV-2 has not been spatially and temporally uniform in an outbreak. With the identification of an infectious disease outbreak, recognizing and evaluating anomalies (excess and decline) of disease incidence spread at the time of occurrence during the course of an outbreak is a logical next step.

We propose and formulate a hypergeometric probability model that investigates anomalies of infectious disease incidence spread at the time of occurrence in the timeline for many geographically described populations (e.g., hospitals, towns, counties) in an ongoing daily monitoring process. It is structured to determine whether the incidence grows or declines more rapidly in a region on the single current day or the most recent few days compared to the occurrence of the incidence during the previous few days relative to elsewhere in the surveillance period. The new method uses a time-varying baseline risk model, accounting for regularly (e.g., daily) updated information on disease incidence at the time of occurrence, and evaluates the probability of the deviation of particular frequencies to be attributed to sampling fluctuations, accounting for the unequal variances of the rates due to different population bases in geographical units.

We attempt to present and illustrate a new model to advance the investigation of anomalies of infectious disease incidence spread by analyzing subsamples of spatiotemporal disease surveillance data from Taiwan on dengue and COVID-19 incidence which are mosquito-borne and contagious infectious diseases, respectively. Efficient R programs for computation are available to implement the two approximate formulae of the hypergeometric probability model for large numbers of events.

## Introduction

The spread of infectious disease is often time-varying and spatially heterogeneous in the transmission during an outbreak. For Coronavirus disease 2019 (COVID-19) - caused by the severe acute respiratory syndrome coronavirus 2 (SARS-CoV-2) - in which cases seem to surge and spread abruptly in time and space, it is essential to devise sensitive and efficient procedures for characterizing and assessing the spread of disease occurrence at the time of occurrence on an ongoing basis. Variability in disease incidence patterns during emerging or resurging infectious disease outbreaks can provide context to elucidate factors that govern current disease activity and epidemiologic transmission and inform strategies for epidemic control, prevention, and forecasts. The problem of recognizing and evaluating geographical variability in incidence spread at the time of occurrence for ongoing space-time infectious disease surveillance, as in COVID-19 or similar infectious diseases, is described and presented in this report.

One major application of spatial and temporal statistics is in epidemiology, in particular, characterizing spatial and temporal patterns of observed disease incidence and mortality, using existing health data collected on a basis of geographic units such as counties. The objective of chronic and infectious disease surveillance over space and time includes disease outbreak detection, trend monitoring, clustering detection, and spread assessment (Cliff and Ord 1981; Mantel 1967; Robertson and Nelson 2010). What distinguishes between the analytic models for various disease surveillance analyses is their aims and applicability. The spatiotemporal characteristics of chronic diseases like cancer differ in many surveillance aspects from those of infectious diseases. Retrospective disease surveillance analysis generally aims to better understand the disease etiology and underlying causal mechanism or identify a common causal exposure for disease. The importance of prospective statistical disease surveillance methods to detect disease outbreaks, disease clusters, or an increased incidence as early as possible is to minimize morbidity or mortality through the timely implementation of effective disease prevention and control measures (Sonesson and Bock 2003; Woodall 2008).

Most of the statistical methods for spatial and temporal disease surveillance analysis are retrospective, including those used in a temporal series (Jagan et al. 2020; Wallenstein and Neff 1987; Wu et al. 2008; Wu et al. 2010), in a spatial series (Cliff and Ord 1981; Cressie 1992; Cressie and Chan 1989; Grimson et al. 1981; Kulldorff 1997; Lai et al. 2018; Warren et al. 2020; Wu et al. 2021; Wu and Shete 2020), in a time-space series (Ederer et al. 1964; Mantel 1967; Wallenstein et al. 1989; Wu et al. 2008), and in a space-time series (Knox 1964; Kulldorff et al. 1998). Over the past decades, there has been a dramatically increased interest in dealing with prospective disease surveillance methods in the statistical and epidemiological literature (Sonesson and Bock 2003; Unkel et al. 2012; Woodall 2008). Growing prospective statistical methods in disease surveillance exist for early detection of disease outbreaks and active disease clusters (Robertson et al. 2010), including those used in a temporal series (Farrington et al. 1996; Grimson and Mendelsohn 2000; Hutwagner et al. 1997; Naus and Wallenstein 2006; Nobre and Stroup 1994; Reis and Mandl 2003; Wu et al. 2017) and those used in a space-time series (Kulldorff 2001; Kulldorff et al. 2005). These prospective analytic methods in a temporal series generally aim to identify an ongoing disease outbreak or active disease cluster or to signal an increase in the rate of incidence that remains present as early as possible over a broad geographical area (e.g., country) or long temporal scale (e.g., years). They are useful when relatively few cases are observed in any one jurisdiction. They usually require knowledge or assumptions of probability distributions that underlie the data and may need exploratory studies or preliminary analysis to estimate model parameters. The prospective spatial scan statistic in a space-time series (Kulldorff 2001; Kulldorff et al. 2005) is designed to scan thousands or millions of possible geographical candidates and quickly detect emerging geographical disease clusters that remain present during the last time period for which data are available. It was recently used to detect geographical clusters of increasing SARS-CoV-2 test percent positivity in 2020 in New York City, New York, USA (Greene et al. 2021).

The infection rate and spread of the SARS-CoV-2 virus have not been uniform spatially and temporally. For instance, the data often show that COVID-19 cases are growing more rapidly in places while the incidence is declining across the country or, in a number of areas where new cases are declining, the new cases are climbing in many other areas. Our experience with COVID-19 and Severe Acute Respiratory Syndrome (SARS) in 2002 shows the importance of early disease outbreak detection and disease incidence spread assessment in understanding and managing the spread of infectious diseases. However, most of the prospective statistical methods in space-time disease surveillance and the intended functions of many surveillance systems focus on earlier detection of disease outbreaks, disease clusters, or an increase in the incidence that remains present. Few studies have focused on assessing infectious disease spread at the time of occurrence throughout an emerging or resurging outbreak of infectious disease across space. With the identification of an infectious disease outbreak, recognizing and assessing anomalies (excess and decline) of disease incidence spread at the time of occurrence over space is a logical next step.

The purpose of this paper is to propose and develop a statistical method that investigates spatial variability in observed infectious disease incidence patterns at the time of occurrence in a prospective time-space series. We devise a sensitive and efficient procedure for evaluating geographical heterogeneity between disease spread rates for the current time period and surveillance period, which are numbers of days on an ongoing basis, regardless of newly emerging or resurging infectious disease outbreaks like COVID-19. The method aims at near real-time assessment of an important excess of incidence or decline in incidence occurring in a region or several regions combined during the current time period, relative to elsewhere in the region under study. It is structured to determine whether the incidence on the single current day of occurrence or on the most recent few days grows or declines more rapidly in a region or several regions combined, relative to elsewhere, within a surveillance period.

The proposed model contains a stochastic sense that is designed to be sensitive to disease incidence at the time of occurrence, ignores incidence that occurred long ago and is not likely to affect current disease activity, and requires mild assumptions on the basis of random arrangements of epidemiological events. Testing for excessive aggregations of disease incidence that occurred during a single current unit of time (e.g., day, week) or the most recent few consecutive units of time in one region is used to signal the occurrence of an important excess of incidence in the current time period relative to elsewhere, permitting the immediate response and application of early intervention. In contrast, detecting an unusually sparse incidence of disease at the time of occurrence in one region characterizes the current disease activity and epidemiologic transmission in an opposite way. It determines whether an important decline in disease incidence is occurring in places in the current time period, allowing for immediate assessment of an intervention strategy and decisions regarding prevention programs in the ongoing daily monitoring process.

Spatio-temporal analysis of disease incidence anomalies based on raw rates or counts in geographic units, such as counties, can be misleading because distinct geographic units generally have substantially different population bases, e.g., population size, and, correspondingly, have highly unequal variances of the rates (Cressie and Chan 1989; Cressie and Read 1989; Wu and Shete 2020). Recent studies have increasingly shown that the assumption of constant null baseline risk may substantially limit the sensitivity and usefulness of analytical models for spatial or temporal disease surveillance analysis in the statistical and epidemiological literature (Jagan et al. 2020; Naus and Wallenstein 2006; Warren et al. 2020). The prospective statistical model we propose here is methodologically different in several surveillance contextual factors, including the function and scale, from existing methods for prospective disease surveillance analysis, such as the various scan statistics, the CUSUM, and the GLMM (Robertson et al. 2010). Our proposed method has salient features and addresses important problems. In particular:

1. We formulate a hypergeometric probability model to determine whether or not an important growth or decline in incidence occurs to an extent greater than what would be expected by chance variation, adjusting for the unequal variances of the rates in geographic units.
2. Without restricting to the assumption of temporally constant null baseline risk, time-varying baseline risk of disease occurrence is proposed and modeled, accounting for daily updated information on disease incidence at the time of occurrence across space.
3. Two approximate formulae for computation are provided, which can be implemented in efficient R programs for calculation.
4. The method aims to recognize whether the incidence in a region currently progresses at the same rate, at a higher rate, or at a lower rate than the incidence that occurs elsewhere in comparison with the incidence that occurred in the previous few days during the course of an outbreak.

We illustrate the proposed statistical method and investigate geographical heterogeneity in observed infectious disease incidence patterns at the time of occurrence, using subsamples of spatiotemporal disease surveillance data from Taiwan on dengue incidence, a mosquito-borne tropical infectious disease, and COVID-19 incidence, a contagious disease that spreads from person to person. These analyses demonstrate that the proposed method is useful to efficiently evaluate geographical heterogeneity in anomalous infectious disease incidence spread at the time of occurrence in a time-space series. With the global emergence and resurgence of pandemics and epidemics such as COVID-19, Zika, dengue, and chikungunya, statistical methods for anomalies of observed disease incidence spread across space during the course of an outbreak in the ongoing surveillance of infectious diseases are particularly desired and needed.

## Methods

In this section, we introduce our statistical method for prospective infectious disease surveillance in a time-space series and provide formulae and R programs for assessing the statistical significance of geographical heterogeneity in anomalies of disease incidence spread at the time of occurrence during the course of an outbreak as the disease incidence data accumulating over time.

### Exact probability distribution for evaluating spatial heterogeneity in anomalous disease incidence spread

Suppose that C adverse health-related events have occurred over all S areas during T days. Consider the frequency of health-related events that occurred in some area(s) within the most recent w days compared with those in the area(s) and elsewhere in the geographical region under study in the T-w previous days. What interests us is to determine whether an important excess of disease incidence or decline in disease incidence occurred in some area(s) during the current w-day period compared to the incidence in the T-w previous days, relative to elsewhere, within the T-day surveillance period. That is, it recognizes and evaluates whether the incidence in some place in the most recent few days is growing or declining more rapidly relative to elsewhere, compared with the occurrence of the incidence during the previous few days.

Suppose that the spatial-temporal occurrence of events over all S areas in the T-day surveillance period under study is denoted by a rectangular SxT array of the form:

$$\left(\begin{array}{ccc} C_{11} & \cdots & C_{1T}\\ \vdots & \ddots & \vdots \\ C_{S1} & \cdots & C_{ST} \end{array}\right),$$

where $C_{ij}$ is the number of events that occurred in the i-th area and on the j-th day. The total number (C) of observed events over all S areas in the T-day surveillance period is equal to $C\;=\;\sum_{i}\sum_{j}c_{ij}$, where 1≤i≤S,1≤j≤T. When there is no time-space interaction, the expected number of $C_{ij}$ is equal to $\left(C_{i.}C_{.j}\right)∕C$, conditional on the observed row marginal (space domain), column marginal (time domain), and grand totals.

Letting the symbol I denote the current w days or the last w days for which data are available in the time domain, I represents days T, T-1, and T-w+1 combined in the SxT array. In this setting, the number of events that occurred in area k over the T-day surveillance period is denoted by $C_{k.}$, the number of events in area k during the current w-day period, by $C_{kl}$, and the number of events in area k within the T-w previous days, by $C_{k.}\text{-}\;C_{kl}$. Correspondingly, the number of events that occurred outside area k over the T-day surveillance period is $C\;\text{-}\;C_{k.}$, the number of events outside area k during the current w-day period, $C_{.l}\;\text{-}\;C_{kl}$, and the number of events outside area k within the T-w previous days, $\left(C\;\text{-}\;C_{k.})-(C_{.l}\;\text{-}\;C_{kl}\right)$. When the spatial component is divided into area k and outside area k, and the temporal component, into the most recent w days and the T-w previous days, the original SxT array for spatial-temporal occurrence of events is transformed into a 2X2 array of the form:

$$\left(\begin{array}{cc} C_{k.}-C_{kl} & C_{kl}\\ \left(C-C_{k.})-(C_{.l}-C_{kl}\right) & C_{.l}-C_{kl} \end{array}\right).$$

Assuming that $C_{kl}$ is the random variable that represents the number of events occurring within the most recent w days in area k and that there is no time-space interaction, $C_{kl}$ is distributed as a hypergeometric distribution with $\text{mean}\;=\;\left(C_{k.}\times C_{.l}\right)∕C$, conditional on the observed margins, and probability function given by

(1) $$P(C_{kl}=c_{kl}∕\;C,\;c_{k.},\;c_{.l})=\left(\begin{array}{c} c_{k.}\\ c_{kl} \end{array}\right)\left(\begin{array}{c} C-c_{k.}\\ c_{.l}-c_{kl} \end{array}\right)∕\left(\begin{array}{c} C\\ c_{.l} \end{array}\right),\;c_{kl}=0,\;1,\;2,\;.\ldots \;,\;c_{.l}$$

where $c_{kl}$ is the observed number of events within the most recent w days in area k.

The proposed statistical method for prospective infectious disease surveillance is based on this random variable $C_{kl}$. Statistical power and sensitivity of our method are based on the fact that if spatially related cases are to excessively aggregate within the most recent w days in area k, the observed number of $C_{kl}$ tends to be large. In contrast, the other cases tend to have a larger average separation in the rest of the areas under study in the surveillance period.

The p-value form, $P(C_{kl}\geq c_{kl}∕\;C,\;c_{k.},\;c_{.l})$, of our proposed method from Expression (1) is used to measure an empirical growth of incidence in area k within the most recent days in comparison with the occurrence of the incidence during the previous few days, relative to elsewhere, in the T-day surveillance period. A small probability of $P(C_{kl}\geq c_{kl}∕\;C,\;c_{k.},\;c_{.l})$ indicates that the occurrence of $c_{kl}$ events within the most recent w days in area k, compared with the frequency of events occurring during the T-w previous days, excessively aggregates and represents an important excess of disease incidence relative to elsewhere in the geographical region under study. That is, the incidence currently grows more rapidly in area k relative to elsewhere in the T-day surveillance period.

In contrast, the probability of $P(C_{kl}\leq c_{kl}∕\;C,\;c_{k.},\;c_{.l})$ characterizes opposite aspects of an observed spatiotemporal disease incidence pattern and is used to measure an empirical decline of incidence within the most recent days. A small probability of $P(C_{kl}\leq c_{kl}∕\;C,\;c_{k.},\;c_{.l})$, from Expression (1) indicates that the observed $c_{kl}$ events occurring within the most recent w days in area k are empirically sparse and represent an important decline in disease incidence in comparison with the incidence occurred within the previous few days relative to elsewhere. The incidence in area k currently declines more rapidly than elsewhere.

### Approximate formulae and R programs for hypergeometric function computation

The exact probability of Expression (1) and its p-value forms for large numbers of C, $C_{k.}$, $C_{.l}$, and $C_{kl}$ can be computationally intensive. We suggest the use of two approximate formulae for computation in this report. The first approximate formula is a continuation formula of the hypergeometric functions, based on the hypergeometric differential equation (Bühring 1987), and can be implemented in a package “hypergeo” of the Statistical Package R version 4.2.1 (R_Foundation 2023) by Robin K. S. Hankin (https://functions.wolfram.com/PDF/Hypergeometric2F1.pdf). The second one uses a normal approximation for cumulative hypergeometric probabilities (Molenaar 1973):

$$P(C_{kl}\leq c_{kl})≑\Phi \{{2C}^{-1∕2}[{\left(c_{kl}+0.75\right)}^{1∕2}{\left(C-c_{k.}-c_{.l}+c_{kl}+0.75\right)}^{1∕2}-(c_{.l}-c_{kl}-\;\;{0.25)}^{1∕2}{\left(c_{k.}-c_{kl}-0.25\right)}^{1∕2}]\}.$$

This relatively simple approximation has been shown to be considerately accurate by extensive empirical studies (Johnson et al. 1992; Ling and Pratt 1984). This report used both approximate formulae to compute hypergeometric probabilities using the Statistical Package R version 4.2.1 (R_Foundation 2023).

### Time-varying baseline risk model

In the time domain, let $Y_{T}(t)$ be the observed number of adverse health events occurring within the T-day surveillance period at a current time of t. That is, $Y_{T}(t)$ is the frequency of events occurring within the T consecutive days, t−T+1, t−T+2,…,t. Similarly, the frequency of events during the current w-day period at time t, denoted by $Y_{w}(t)$, is the frequency of events during the current w consecutive days, t−w+1, t−w+2,…,t. For given values of T and w, the current time period and surveillance period shift each day and remain to be T and w consecutive days, respectively, as their start day and the end day move by an increment of 1 day simultaneously. The rectangular SxT array for spatial-temporal occurrence of events at a current time of t, t+1, and t+2, is respectively $\left(C^{t-T+1},C^{t-T+2},\cdots ,C^{t}\right)$, $\left(C^{t-T+2},C^{t-T+3},\cdots ,C^{t+1}\right)$, and $\left(C^{t-T+3},C^{t-T+4},\cdots ,C^{t+2}\right)$, where $C^{i}$, the ith column of an SxT array, represents the spatial occurrence of events at time i across all S areas.

In this setting, the proposed method can be performed for daily analysis of geographical heterogeneity in anomalous infectious disease incidence spread at a current time of t, t+1, t+2,.… with $Y_{T}(t)$ and $Y_{w}(t)$; $Y_{T}(t+1)$ and $Y_{w}(t+1)$; $Y_{T}(t+2)$ and $Y_{w}(t+2),.\ldots$, respectively, as the incidence data update daily in an ongoing space-time disease surveillance. This design would permit our proposed model to be sensitive to the current or most recent state of an observed disease incidence pattern and ignore disease incidence that occurred long ago and is not likely to affect current disease transmission activities.

The modeling of time-varying baseline risk of disease occurrence is based on the values of C, $c_{k.}$, $c_{.l}$, and $c_{kl}$ in Expression (1), which vary daily with the corresponding spatial-temporal occurrence of events in the SxT array at a current time of t, t+1, t+2,……, accounting for daily updated information on disease incidence at the time of occurrence across space. Spatially or temporally varying distributions and patterns of disease occurrence often have a profound influence on analysis. Recent studies have shown that the assumption of constant null baseline risk may substantially limit the sensitivity and usefulness of analytical models for spatial or temporal disease surveillance analysis in the statistical and epidemiological literature (Jagan et al. 2020; Naus and Wallenstein 2006; Warren et al. 2020).

## Applications of Hypergeometric Models to Data of Dengue and COVID-19 Outbreaks

We analyzed subsamples of spatio-temporal surveillance data and investigated geographical heterogeneity in the observed infectious disease spread of dengue fever in 2015 and COVID-19 in 2022 in Taiwan using our proposed method. Information on dengue and COVID-19 cases collected in Taiwan is publicly available through the Taiwan Centers for Disease Control (https://www.cdc.gov.tw/En/Category/NewsPage/bg0g_VU_Ysrgkes_KRUDgQ) and the Taiwan Government Open Data (https://data.cdc.gov.tw/en/dataset/) websites. This information includes the date an individual was diagnosed with infection, his or her residence at diagnosis, place of infection, gender, and age. A nominal significance level of 0.05 is used to determine whether an important excess or decline in incidence occurs in a region at the time of disease occurrence relative to elsewhere.

### Applications to Dengue Outbreak Data

Dengue fever is the most common arbovirus infection in humans, with viral transmission occurring in more than 100 countries in tropical regions. It is estimated that 390 million dengue infections occur annually, of which 50–100 million cases have apparent clinical manifestations (Bhatt et al. 2013; Stanaway et al. 2016). Dengue is a notifiable communicable disease in Taiwan. The study population used for this investigation is patients with laboratory confirmed autochthonous dengue infection, which thus excludes imported cases of dengue. The largest dengue outbreak in Taiwan since World War II occurred in 2015. There were 43,419 confirmed autochthonous cases in Taiwan in 2015, among which 53% (22,842 cases) occurred in Tainan City, located in the southern tropical region of Taiwan (Lai et al. 2018).

**Figure 1:**
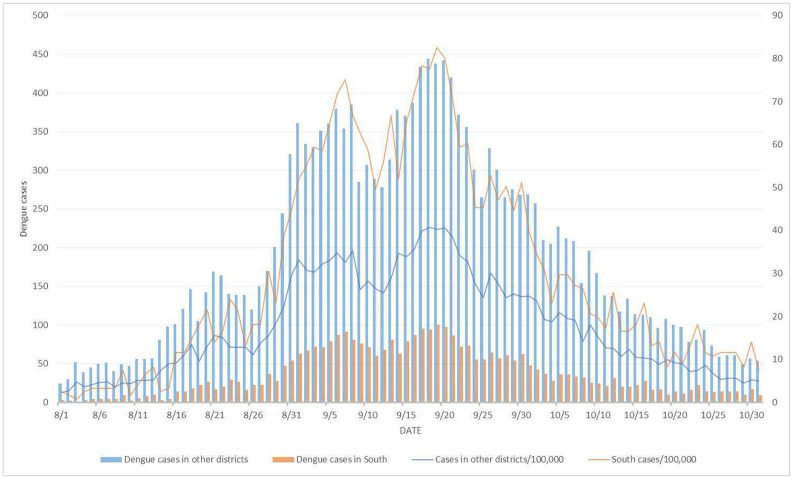
Daily dengue incidence data for South District, Tainan City, Taiwan, from August to October 2015.

We selected a subsample of dengue incidence data in Tainan and analyzed a time-space series of data from August to October 2015. The rates, which were the numbers of dengue cases per 100,000 persons, ranged from 0 to 4,497 among the 37 districts in Tainan in 2015. A district is an administratively defined subdivision of a city in Taiwan and has its own health department that regularly reports health information to the city government. The 11 districts with the highest rates were West Central (rate of 4,497), North (4,313), South (2,785), East (1,673), Anping (1,401), Yongkang (1,159), Annan (984), Yujing (480), Rende (422), Xinhua (358), and Guiren (315). The remaining 26 districts had a rate of 202 or less (Lai et al. 2018). Figure 1 displays the daily dengue incidence data for Tainan's South District and the combined incidence in the remaining 10 districts with the highest dengue incidence rates from August to October 2015. District-specific dengue incidence intensity map in 2015 Tainan can be found in Figure 3 of our previous report (Lai et al. 2018).

We used historical data on dengue fever to mimic a prospective space-time disease surveillance system with daily analyses from August to October 2015. For each of these days, the analysis only used data prior to and including the day in question, ignoring all data from subsequent days. We illustrate the use of Expression (1) and its p-value forms for near real-time assessment of excessive aggregations or decline of incidence in South District during the most recent few days in comparison with the incidence in the previous few days relative to the other 10 districts with the highest dengue rates combined in Tainan.

Where a day is the unit of time, setting w=3 and T=10, the number of cases reported during the most recent 3 days is compared with the number of cases in the previous 7 days between South District and the other 10 districts combined. C adverse health events have occurred over all 11 districts during 10 days in this setting. $C_{k.}$ denotes the number of events in South District during the 10-day surveillance period; $C_{kl}$, the number of events within the current 3-day period in South District; and $C_{.l}$, the number of events within the current *3*-day period over all 11 districts in the 2X2 array for spatial-temporal occurrence of events and Expression (1).

This report used both approximate formulae to compute p-values, using the Statistical Package R version 4.2.1 (R_Foundation 2023). The p-values for excessive aggregations of incidence during the most recent 3 days in South District, relative to the other 10 districts combined, on August 18-22 in the 10-day surveillance period are shown as follows (probability based on the first approximation / probability based on the second approximation):

August 18: $P(C_{kl}\geq 46|C=899,\;C_{.l}=414,\;C_{k.}=87)=0.1095∕0.0735$,

August 19: $P(C_{kl}\geq 54|C=968,\;C_{.l}=426,\;C_{k.}=100)=0.0221∕0.0134$,

August 20: $P(C_{kl}\geq 66|C=1087,\;C_{.l}=459,\;C_{k.}=124)=0.0058∕0.0035$,

August 21: $P(C_{kl}\geq 65|C=1212,\;C_{.l}=481,\;C_{k.}=136)=0.0258∕0.0178$,

August 22: $P(C_{kl}\geq 63|C=1332,\;C_{.l}=538,\;C_{k.}=148)=0.3132∕0.2607$.

Low p-values on August 19-21 indicate that an important excess of dengue incidence in South District during the most recent 3 days, relative to the other 10 districts with the highest dengue rates combined, is identified at a nominal significance level of 0.05 within the 10-day surveillance period. That is, the dengue incidence spreads more rapidly in South District in the most recent 3-day period daily compared to the incidence that occurred during the previous few days relative to elsewhere within the 10-day surveillance period.

Next, we illustrate the use of Expression (1) and its p-value form to evaluate the evidence of an important decline in dengue incidence at the time of occurrence in South District in comparison with the occurrence of the incidence during the previous few days, relative to the other 10 districts combined in the daily monitoring process.

September 14: $P(C_{kl}\leq 212|C=4086,\;C_{.l}=1182,\;C_{k.}=757)=0.2832∕3425$,

September 15: $P(C_{kl}\leq 223|C=4096,\;C_{.l}=1285,\;C_{k.}=757)=0.1122∕0.1120$,

September 16: $P(C_{kl}\leq 229|C=4104,\;C_{.l}=1364,\;C_{k.}=757)=0.0290∕0.0287$,

September 17: $P(C_{kl}\leq 261|C=4188,\;C_{.l}=1452,\;C_{k.}=761)=0.4229∕0.4234$,

September 18: $P(C_{kl}\leq 276|C=4260,\;C_{.l}=1541,\;C_{k.}=774)=0.3875∕0.3879$.

On the 16^th^ of September, a low p-value of 0.0290/0.0287 for current 3-day paucity of incidence is obtained, indicating that an important decline in dengue incidence during the current 3-day period has occurred in South District, relative to the other 10 districts combined within the 10-day surveillance period. Low dengue incidence in South District on September 14-16, compared with the incidence that occurred during the previous few days, results in a small p-value on September 16^th^, indicating that the dengue incidence in South District currently declines faster relative to elsewhere within the 10-day surveillance period.

### Applications to COVID-19 Outbreak Data

COVID-19 has become a global pandemic, with several million confirmed COVID-19-related deaths worldwide. Numerous variants of SARS-CoV-2 are tracked globally during this pandemic. Heart disease and cancer were the leading causes of death in the United States, followed by COVID-19, from March 2020 to October 2021. During the 20-month period studied, COVID-19 accounted for 1 in 8 deaths (or 697,000 deaths). The pandemic may also have indirectly led to increases in other causes of death, including heart disease, diabetes, Alzheimer’s disease, and unintentional injuries (Shiels et al. 2022).

**Figure 2:**
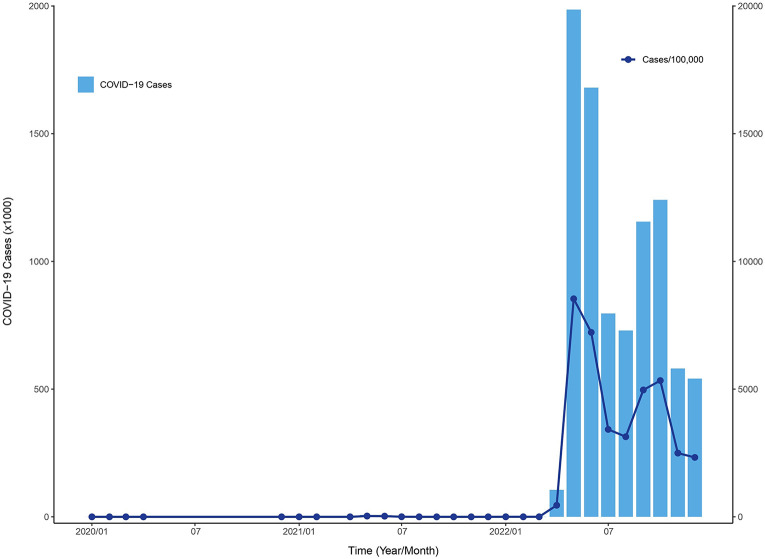
Monthly COVID-19 incidence distribution in Taiwan through December 2022

**Figure 3:**
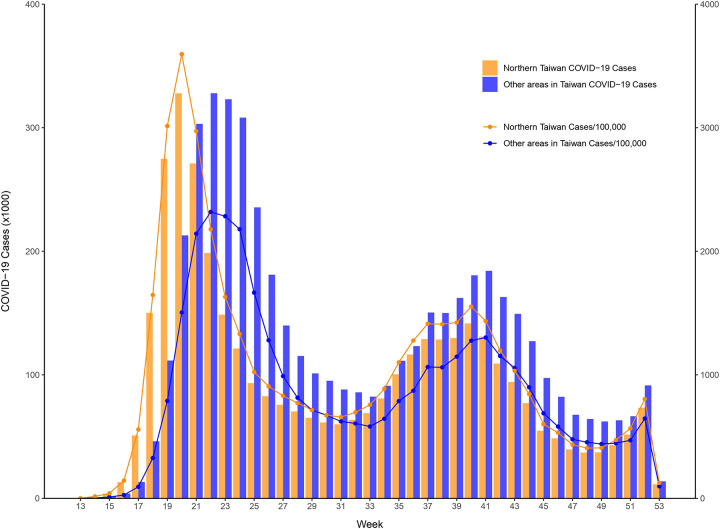
Weekly COVID-19 incidence distributions for northern Taiwan and elsewhere between April and December 2022

**Figure 4:**
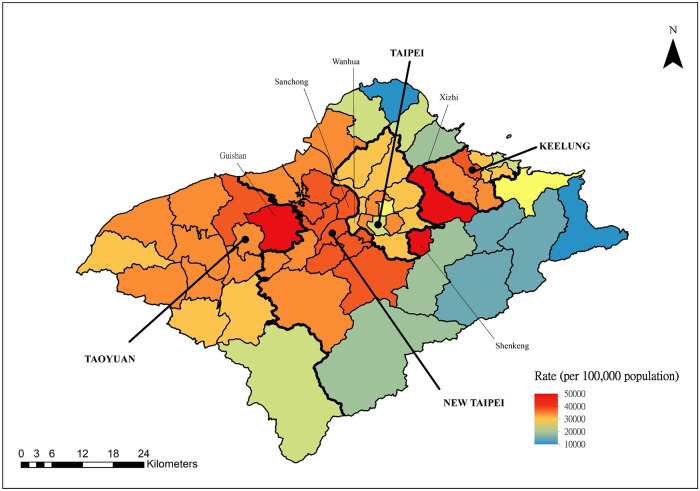
City- and district-specific COVID-19 incidence intensity map in northern Taiwan from April to December 2022

The number of confirmed autochthonous COVID-19 cases in Taiwan exceeded 8.8 million from January 2020 to December 2022, with a rate of more than 37 thousand per 100,000 persons. Figure 2 shows the monthly COVID-19 incidence distribution that occurred through December 2022. The vast majority of COVID-19 cases occurred after April 2022. This largest outbreak started in northern Taiwan, which consists of 4 cities, Taipei, New Taipei, Taoyuan, and Keelung, with 12, 29, 13, and 7 districts, respectively. The weekly COVID-19 incidence distributions for northern Taiwan and elsewhere between April and December 2022 are presented in Figure 3. The city- and district-specific COVID-19 incidence intensity map in northern Taiwan from April to December 2022 is displayed in Figure 4.

**Figure 5:**
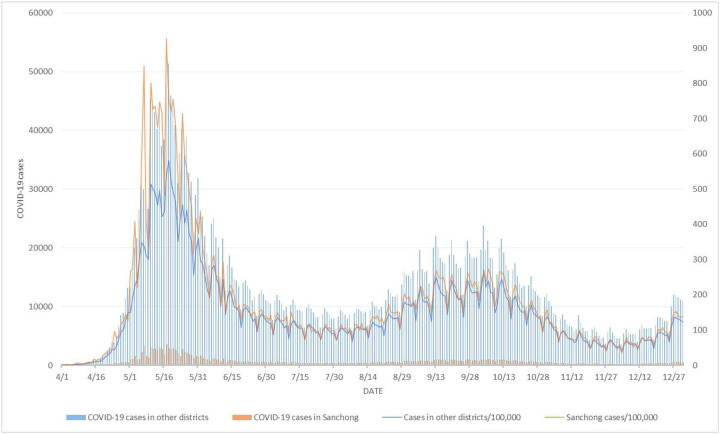
Daily COVID-19 incidence data for Sanchong District as well as the combined incidence in the other 60 districts in northern Taiwan from April to December 2022

We, again, applied the proposed method by mimicking daily prospective analyses of COVID-19 incidence data for northern Taiwan from April 2022, using data prior to and including the day in question and ignoring all data from subsequent days. Sanchong District of New Taipei City was one of the hardest hit districts during the earlier stage of the outbreak. Figure 5 displays the daily COVID-19 incidence data for Sanchong District as well as the combined incidence in the other 60 districts in northern Taiwan from April to December 2022. Using Expression (1), the p-values for evaluating the evidence of an important decline in COVID-19 incidence during the most recent 3 days in Sanchong District relative to the other 60 districts combined on July 13-22 in the 10-day surveillance period are shown as follows:

July 13: $P(C_{kl}\leq 1533|C=107395,C_{k.}=4981,C_{.l}=33207)=4.18\times 10^{-1}∕4.18\times 10^{-1}$,

July 14: $P(C_{kl}\leq 1376|C=105700,C_{k.}=4881,C_{.l}=32153)=2.60\times 10^{-4}∕2.50\times 10^{-4}$,

July 15: $P(C_{kl}\leq 1319|C=102945,C_{k.}=4741,C_{.l}=30288)=6.75\times 10^{-3}∕6.65\times 10^{-3}$,

July 16: $P(C_{kl}\leq 1255|C=100884,C_{k.}=4654,C_{.l}=29147)=1.50\times 10^{-3}∕1.46\times 10^{-3}$,

July 17: $P(C_{kl}\leq 1127|C=96561,C_{k.}=4457,C_{.l}=26070)=4.22\times 10^{-3}∕4.13\times 10^{-3}$,

July 18: $P(C_{kl}\leq 1096|C=96840,C_{k.}=4378,C_{.l}=26426)=2.95\times 10^{-4}∕2.81\times 10^{-4}$,

July 19: $P(C_{kl}\leq 1129|C=97173,C_{k.}=4283,C_{.l}=27706)=7.00\times 10^{-4}∕6.74\times 10^{-4}$,

July 20: $P(C_{kl}\leq 1204|C=100014,C_{k.}=4279,C_{.l}=30925)=2.66\times 10^{-5}∕2.49\times 10^{-5}$,

July 21: $P(C_{kl}\leq 1195|C=98586,C_{k.}=4104,C_{.l}=30217)=1.52\times 10^{-2}∕1.50\times 10^{-2}$,

July 22: $P(C_{kl}\leq 1127|C=95516,C_{k.}=3978,C_{.l}=27977)=8.97\times 10^{-2}∕8.96\times 10^{-2}$.

Small p-values for current 3-day paucity of incidence on July 14-21 presented above show that an important decline in COVID-19 incidence during the most recent 3 days is detected in Sanchong District each of these days relative to the other 60 districts combined in northern Taiwan over the 10-day surveillance period. The incidence in Sanchong District declined more rapidly relative to elsewhere in northern Taiwan daily on July 14-21.

Next, we evaluated the evidence of excessive aggregations of COVID-19 incidence in Sanchong District relative to the other 60 districts combined in the daily monitoring process. The p-values for current 3-day excess of incidence in Sanchong District relative to elsewhere in the 10-day surveillance period on September 15-19 are presented as follows:

September 15: $P(C_{kl}\geq 2942|C=181516,C_{k.}=8250,C_{.l}=63361)=7.25\times 10^{-2}∕7.02\times 10^{-2}$,

September 16: $P(C_{kl}\geq 2868|C=179493,C_{k.}=8344,C_{.l}=58783)=6.61\times 10^{-4}∕6.37\times 10^{-4}$,

September 17: $P(C_{kl}\geq 2795|C=180567,C_{k.}=8411,C_{.l}=55991)=3.83\times 10^{-6}∕3.77\times 10^{-6}$,

September 18: $P(C_{kl}\geq 2449|C=177339,C_{k.}=8296,C_{.l}=50097)=4.50\times 10^{-3}∕4.37\times 10^{-3}$,

September 19: $P(C_{kl}\geq 2391|C=180195,C_{k.}=8437,C_{.l}=51315)=6.17\times 10^{-1}∕6.11\times 10^{-1}$.

Small p-values for current 3-day excess of incidence in Sanchong District relative to the other 60 districts combined were obtained on September 16-18, indicating that the COVID-19 incidence in Sanchong District spreads faster relative to elsewhere daily on those days.

## Discussion

The spread of a virus such as SARS-CoV-2 is not uniform temporally and spatially in an outbreak. With the identification of an infectious disease outbreak, it is logical and essential to characterize anomalous disease incidence patterns in space and time during the course of an outbreak. In this report, we proposed and formulated a hypergeometric probability model that investigates anomalies of infectious disease incidence spread at the time of occurrence in the timeline for many geographically described populations (e.g., hospitals, towns, counties) in an ongoing daily monitoring process. This method is useful to recognize and evaluate geographical heterogeneity in observed infectious disease incidence spread in a prospective time-space series. Our proposed method aims to evaluate the evidence of an important excess of incidence or decline in incidence occurring in a region during the current time period relative to elsewhere in the geographical region under study in a near real-time manner.

For disease mapping, mapping the raw rates or counts of incidence based on geographic units, such as counties, can be misleading. One main reason is that geographic units generally have substantially different population bases, such as population size. Geographic units with a small population base typically have larger variances for the rates and, correspondingly, are more likely to exhibit their rates that fluctuate greatly from the unknown actual rate. Appropriate probability distributions or transformations, such as the Freeman-Tukey square-root transformation, can be used to adjust for or stabilize the unequal variances of the rates in geographic units (Cressie and Chan 1989; Cressie and Read 1989; Freeman and Tukey 1950; Wu et al. 2021). We developed a Poisson probability model that assesses the statistical significance of spatial disease incidence anomalies, accounting for the unequal variances of the county rates with the adjustment of covariates that are known or hypothesized disease risk factors in our previous report (Wu and Shete 2020). Here, we proposed and formulated a hypergeometric probability model for evaluating the probability of the deviation of a particular frequency to be attributed to sampling fluctuations, which provides a crude way to account for the unequal variances of the district rates.

As noted by recent articles in the statistical and epidemiological literature, the assumption of constant null baseline risk may substantially limit the sensitivity and usefulness of analytical models for spatial or temporal disease surveillance analysis (Jagan et al. 2020; Naus and Wallenstein 2006; Warren et al. 2020). In response, we proposed a time-varying baseline risk model of disease occurrence, accounting for regularly (e.g., daily) updated information on disease incidence at the time of occurrence across space. In addition, our method contains a stochastic sense that is sensitive to disease incidence at the time of occurrence, ignores incidents that occurred long ago, and requires mild assumptions based on random arrangements of epidemiological events. The methods with these features have more power to detect disease clusters in incidence at the time of occurrence with a duration of one or more days during an ongoing daily data collection and monitoring process (Grimson and Mendelsohn 2000; Wu et al. 2017) than the methods to be retrospectively applied, such as the scan test (Hryhorczuk et al. 1992).

In this study, we attempt to present and illustrate a new statistical model to advance the investigation of anomalies of infectious disease incidence spread at the time of occurrence in the timeline in a prospective time-space series by analyzing subsamples of spatiotemporal disease surveillance data on dengue and COVID-19 incidence from the Taiwan Centers for Disease Control. Our method is designed to focus on the times and areas of both excess and paucity of epidemiologic events at the time of occurrence. Health authorities and epidemiologists may expand (or change) an intervention strategy as soon as a decline in incidence is (or is not) detected after using certain intervention applications in a given region. When an important excess of disease incidence in a region is identified at a time point, response and intervention can be initialized immediately. Diseases for which activity and transmission are affected by environmental or climatic factors are particularly modifiable by intervention.

The exact probability of our proposed hypergeometric probability model can be computationally intensive for large numbers of events. We suggest two approximate formulae for computation. These two approaches can be implemented in the Statistical Package R version 4.2.1 programs (R_Foundation 2023). Both approximate formulae and the corresponding R programs were applied and presented in this study. Our analyses show that these R programs are efficient for calculation.

The global emergence of the SARS-CoV-2 virus, as well as Zika virus infection and its severe forms, Guillain-Barre syndrome and microcephaly, which have been associated with the Zika virus in French Polynesia and Brazil in 2015 (Musso and Gubler 2016), indicates that infectious diseases are a severe global public health problem. As predicted by the U.S. National Institute of Allergy and Infectious Diseases, National Institutes of Health, in 2017, we will inevitably face the challenges of unanticipated infectious disease outbreaks (Paules et al. 2017). Health authorities and epidemiologists must learn through these experiences regarding optimal response to infectious disease threats. Statistical methods to accurately and efficiently determine whether an important excess of incidence or decline in incidence is happening in a region at the time of disease occurrence for ongoing space-time infectious disease surveillance, as presented here, are increasingly desired in light of the recent global emergence of COVID-19 infection.
